# Extending the Spectrum of Dysgraphia: A Data Driven Strategy to Estimate Handwriting Quality

**DOI:** 10.1038/s41598-020-60011-8

**Published:** 2020-02-21

**Authors:** Thibault Asselborn, Mateo Chapatte, Pierre Dillenbourg

**Affiliations:** CHILI Lab, EPFL, Route Cantonale, 1015 Lausanne, Switzerland

**Keywords:** Machine learning, Health care, Signs and symptoms

## Abstract

This paper proposes new ways to assess handwriting, a critical skill in any child’s school journey. Traditionally, a pen and paper test called the BHK test (Concise Evaluation Scale for Children’s Handwriting) is used to assess children’s handwriting in French-speaking countries. Any child with a BHK score above a certain threshold is diagnosed as ‘dysgraphic’, meaning that they are then eligible for financial coverage for therapeutic support. We previously developed a version of the BHK for tablet computers which provides rich data on the dynamics of writing (acceleration, pressure, and so forth). The underlying model was trained on dysgraphic and non-dysgraphic children. In this contribution, we deviate from the original BHK for three reasons. First, in this instance, we are interested not in a binary output but rather a scale of handwriting difficulties, from the lightest cases to the most severe. Therefore, we wish to compute how far a child’s score is from the average score of children of the same age and gender. Second, our model analyses dynamic features that are not accessible on paper; hence, the BHK is useful in this instance. Using the PCA (Principal Component Analysis) reduced the set of 53 handwriting features to three dimensions that are independent of the BHK. Nonetheless, we double-checked that, when clustering our data set along any of these three axes, we accurately detected dysgraphic children. Third, dysgraphia is an umbrella concept that embraces a broad variety of handwriting difficulties. Two children with the same global score can have totally different types of handwriting difficulties. For instance, one child could apply uneven pen pressure while another one could have trouble controlling their writing speed. Our new test not only provides a global score, but it also includes four specific score for kinematics, pressure, pen tilt and static features (letter shape). Replacing a global score with a more detailed profile enables the selection of remediation games that are very specific to each profile.

## Introduction

Despite the entry of the educational system into the digital age, handwriting remains a central skill which must be acquired for every school child since it is still the basis of many core activities such as taking notes, composing stories and self-expression^1–3^. Handwriting is a complex task involving many skills such as attention, perceptual, linguistic and fine motor skills^4–7^. For this reason, even a typically developing child learns handwriting over a period of 10 years, between the ages of 5 and 15^8,9^.

Even with proper training, up to 25% of school children never manage to learn the skill of handwriting^10,11^. Facing increasing cognitive demands upon reaching higher grades, these children are rapidly unable to face simultaneous demands in terms of handwriting, composition and orthography, leading to more general difficulties. For this reason, research has shown a clear correlation between handwriting problems and self-esteem and behavioral and academical development^12^. Hence, it is of prime importance to detect handwriting difficulties as soon as possible in order to begin remediation^13,14^.

Detecting handwriting problems is not a new challenge since the first tests designed to do so were invented at the beginning of the twentieth century^15,16^. The first test was invented by Thorndike^15^ in 1910, constituting a very important contribution “not only to the experimental pedagogy but to the entire movement for the scientific study of education^16^”. Thorndike himself compared its invention to the invention of the thermometer: “Just as it was impossible to measure temperature beyond the very hot, hot, warm, cool, etc., of subjective opinion, so it had been impossible to estimate the quality of handwriting except by such vague standards as one’s personal opinion that given samples were very bad, bad, very good, etc.”^16^. Since then, two main approaches have been used to evaluate handwriting. The first one is a global holistic approach which evaluates handwriting as a whole, while the second one relies on several predefined criteria^17^. The global holistic approach appeared first chronologically and is now virtually obsolete^15,16^ since it relies on subjective judgments. The idea behind this approach is to compare children’s handwriting to typical, pre-sorted samples. The second approach is currently used worldwide in many forms to detect handwriting problems developed in many alphabets. This approach relies on several predefined criteria (rule-based) (e.g., letter size, line straightness, shakiness, and so on), making it more objective than the global approach. As typical examples of this approach, consider the *Hebrew Handwriting Evaluation*^18^ for the Hebrew alphabet, the *Concise Evaluation Scale for Children’s Handwriting* (BHK)^19^, the gold standard test for the Latin alphabet.

The emergence of digital tablets in the last decades opened a new world for the analysis of handwriting problems. Indeed, digital tablets allow researchers to consider not only on the static, final aspects of handwriting (as is currently done) but also the dynamics of handwriting, which have been found to be paramount in the analysis of handwriting disorders^20,21^. In addition, combining digital tablets with machine learning algorithms allows online and objective analyses of handwriting, which is far removed from the time- consuming, hand-written (and thus subjective) analyses done by a therapist. For this reason, some digital tablet-based tests are slowly starting to appear^21–23^. For example, Pagliarini *et al*.^24^ used digital tablets to record data measuring handwriting ability prior to handwriting being performed automatically. They used quantitative models to find patterns that can indicate future writing impairments at a very early age. Mekyska *et al*.^22^ used a Random Forest model to detect dysgraphia. Fifty-four third-grade Israeli children were included in the study, which used a ten-item questionnaire designed to indicate Hebrew handwriting proficiency^25^. Models exploiting digital tablets and machine learning can even be used for the adult population. In^26^, for example, Drotár *et al*. used automatic handwriting assessment tools to help detect Parkinson’s disease.

In the model of interest for this study, Asselborn *et al*.^21^ used 53 features to describe handwriting that were then sorted into four categories, namely, the static (which can possibly be measured with a pen/paper test), kinematic, pressure and tilt (described in the *Method* Section) categories. These features, designed in collaboration with therapists, were then used as input into a Random Forest classifier, which then detected severe handwriting difficulties (dysgraphia) with remarkable accuracy.

Hence, it is quite obvious that the tools currently used to diagnose handwriting difficulties need to be adapted to the digital world. Built on previous research into the classification of dysgraphia^21–23^, we propose, in this paper, a modernized test to evaluate handwriting in a new way. In particular, our method provides a multidimensional analysis of handwriting on different numeric scales, thus allowing handwriting difficulties to be diagnosed more precisely.

In this paper, we present the different steps followed to design our scales. In particular, we describe the process used to model the complexity of our multidimensional variables (features) and their interactions with a data-driven approach, in contrast to the rule-based, simplistic models used in the current handwriting tests. We then discuss the results, advantages and limitations of our scales and how they can be of possible interest to therapists, school teachers and parents.

## Results

Every child involved in this study was asked to write the five first sentences used in the BHK test on a digital tablet (iPad). With the data extracted (x and y coordinates and well as the pressure, tilt and azimuth angle of the pen), we then computed, for each child, a set of 63 features describing their handwriting.

### From the feature value to the feature score

The features describing handwriting and the interactions of these features with grade and age are very complex.

**Figure 1:**
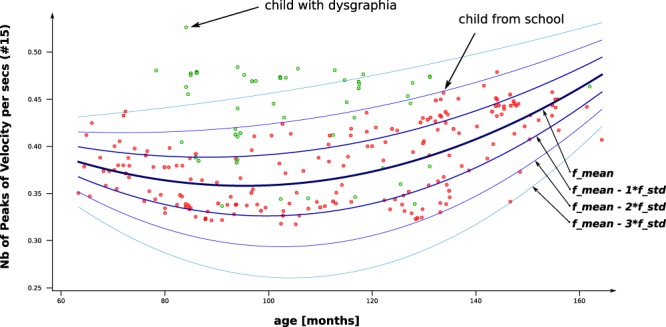
The value of the feature: *Number of Peaks in Velocity per second* (#15) as a function of age is plotted for all individuals in our database. The red points represent children recruited in schools, while the green points represent children with dysgraphia. The interpolated function representing the mean (*f*_*mean*_) is plotted as well as the interpolated function representing the standard deviation (*f*_*std*_). Notice that the points for the children with dysgraphia seem to be located further from the mean compared to the points for their peers.

When computing the features from a handwriting sample, we obtain a range of values that cannot be interpreted directly since they measure handwriting characteristics that vary as functions of a child’s age and gender. The handwriting of a five- year-old child will be obviously very different than the handwriting of a 12-year-old. Handwriting also differs by sex^27,28^. Although current handwriting tests do assess handwriting by grade, we propose to extend this concept by treating age as a continuous variable since it is evident that handwriting evolves at a more rapid pace than annually. To do so, we used a third-degree polynomial function to interpolate each feature for both genders separately. The means (*f*_*mean*_) and standard deviations (*f*_*std*_) of the features as a function of age are provided in Fig. 1 (for a given feature). Hence, a score between 0 and 1 can be computed for each feature according to the deviation from the average (Z-score) via the following equations. This score takes into account the age and gender of the child.

$$Z-score=\frac{| feature-{f}_{mean}(age)| }{{f}_{std}(age)}score={e}^{-\alpha \ast Z-scor{e}^{\beta }},$$

where *α* and *β* are two positive parameters.

In other words, the score indicates how a feature value differs from the mean for children of the same age and gender, making it easily interpretable for therapists. It should be noted that all the children who participated in this study were recruited from French schools, meaning that they all started to learn handwriting in the same grade. In some European countries, children may start to learn handwriting at a different age (e.g., at the age of 7 instead of 6). Hence, at the same age, it is possible for children to have been exposed to different amounts of training (i.e., a 6-year-old child can have a better handwriting than a 7-year-old child). For this reason, we believe that, in addition to gender, different regressions should be used for different countries.

### Projecting features with the PCA algorithm

As shown in the work conducted by Asselborn *et al*.^21^, the features used in this study have advanced discriminatory power when predicting severe handwriting difficulties (dysgraphia). For example, the kinematic and pressure features were found to be far more important than static or tilt features when predicting severe handwriting difficulties. Hence, a numerical scale for estimating handwriting quality based on these features should take into account this difference in terms of predictive importance. However, the feature importance calculations used by Asselborn *et al*.^21^ are linked implicitly with the design of the BHK test since a feature’s importance is based on its power to detect the severe handwriting difficulties (dysgraphia) diagnosed with the BHK test.

In order to design a scale totally independent of the BHK test (and more generally independent of any existing tests), we used the PCA algorithm to determine the importance of the features to predict handwriting quality in a data-driven way. Indeed, as the PCA algorithm projects features into a lower-dimensional space by maximizing the variance within the database, the transformation associated with it will result in the discovery of the combination of features explaining most of the differences in handwriting in our database from those independently of any current handwriting tests.

**Figure 2:**
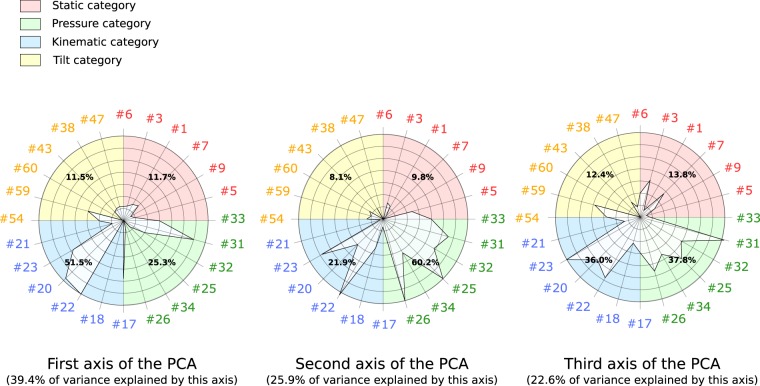
The amount of variability explained by the three first axes of the PCA. The absolute feature importance for each of the three first axes is also represented (in the radar chart) and was sorted according to the four categories. For reasons of clarity, the graphs were plotted with only the six most important features for each category (static, kinematic, tilt and pressure). The features are represented by numbers. The correspondence between the numbers and the feature names and descriptions can be found in the *Method* section.

  Figure 2 shows the amount of variance explained by the three first axes of the PCA algorithm as well as the composition in terms of absolute feature importance sorted into the four categories for the three first axes. For reasons of clarity, we kept only the six most important features for each category (24 in total) to plot the figure.

We can see that kinematic features seems to be the most important in terms of explaining the variability in our database. For instance, the first axis, explaining 39.4% of the variability in our database, is composed of 52.5% kinematic features. The second axis, representing 25.9% of the variability in our database, is composed of a majority (60.2%) of features belonging to the pressure category. Finally, the third axis, representing 22.6% of the variability in our database seems to be mainly composed of kinematic and pressure features. Therefore, the tilt and static features do not appear to be meaningful in explaining handwriting differences between children, at least as opposed to the kinematic and pressure features. The implications of this finding will be explored further in the *Discussion *section.

With regards to the individual features, the *Entropy of Mean of Speed Frequencies* (20), the *Distance of Mean of Speed Frequencies* (22) as well as the *In-Air-Time Ratio* seem to be the most important kinematic features explaining the variability in our database. It is interesting to note that features similar to the two first features were also found to be correlated to handwriting difficulties in previous research^21,22^, while a lot of research has indicated a correlation between the *In-Air-Time Ratio* and handwriting difficulties^22,29,30^.

With regards to the pressure features, the *Standard Deviation of the Pressure* (26), the *Nb of Peaks of Pressure Change per Second* (31) and, to a lesser degree, the *Maximum pressure* (25) seem to be the most important pressure features in terms of explaining the variability in our database. Although the first feature was found to be significantly correlated with handwriting difficulties by Asselborn *et al*.^21^, the other two were not. This is an interesting result since it shows that our method can explain differences in handwriting that are not necessarily associated with handwriting quality (as measured with the BHK test^10^). The implications of this finding will be explored further in the *Discussion* section.

### Unsupervised clustering

**Figure 3:**
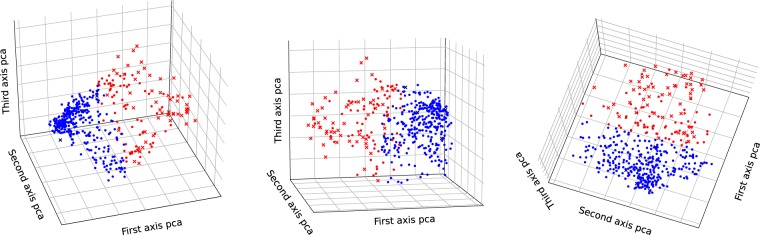
K-Means Algorithm applied to our three-dimensional dataset (defined by the features projected on the three first axes of the PCA), with two clusters under three different projections. The crosses represent children with severe handwriting difficulties (as determined by their BHK scores), whereas the points represent children recruited in schools. The colors represent the two different clusters.

We used a K-means algorithm to cluster the individuals from our database (represented by their features projected with the PCA algorithm) into different groups. For reason of interpretability, we limited the number of cluster to two while hypothesizing that the algorithm would find clusters based on handwriting quality. In Fig. 3, we can see the two clusters (represented by different colors) of individuals projected into the three first axes of the PCA under different projection angles. In order to assess the quality of our clustering, we then checked to see if the dysgraphic children from our database were put into the same cluster. The results showed that around 92% of the children with dysgraphia were clustered together (in the red cluster), while only five children with dysgraphia wound up in the wrong group (blue group). With regards to children without known handwriting difficulties, 86% of them were clustered together (in the blue cluster), while handwriting difficulties were detected in 14% of them (the ones in the red cluster). It is interesting to note that this percentage appears to be comparable to the statistics for French-speaking countries, where approximately 10% of the children in the general population have severe handwriting difficulties (dysgraphia)^19^.

The same process can also be repeated (PCA + K-Means) while taking into account only features from one single category. In such a manner, we obtained a model clustering children according to specific handwriting skills related to a category (static, tilt, pressure and kinematic skills only). We then related these clustering models to the two groups of children present in our database (children with dysgraphia and school children) to see how the models managed to link difficulties related to a specific skill (e.g., kinematic skill) with overall handwriting quality. The results are found in Table 1. When analyzing the results in this table, it is important to realize that the handwriting skills assessed by our categories can be independent. For example, it is possible for children with kinematic deficiencies to have no difficulties with pressure. Hence, it is possible to observe a lower sensitivity value for the "categories" than the "total" skills. However, it is still interesting to analyze the sensitivity of the categories since doing so provides insight into the percentage of children with dysgraphia presenting issues in a specific skill. For instance, 78% of children with dysgraphia present kinematic difficulties, while 58% of them display issues with pressure skills. The implications of this finding will be explored in the *Discussion* section.

**Table 1:** Results of different K-Means clustering models when sub-features belonging to a given category are used (*Total, Kinematic, Pressure, Tilt and Static*) compared to the labeling in our database (children with dysgraphia vs school children).

Category	Sensitivity	Specificity
Total	0.91	0.90
Kinematic	0.78	0.82
Pressure	0.58	0.72
Tilt	0.45	0.68
Static	0.36	0.76

### From a binary to a numerical scale

**Figure 4:**
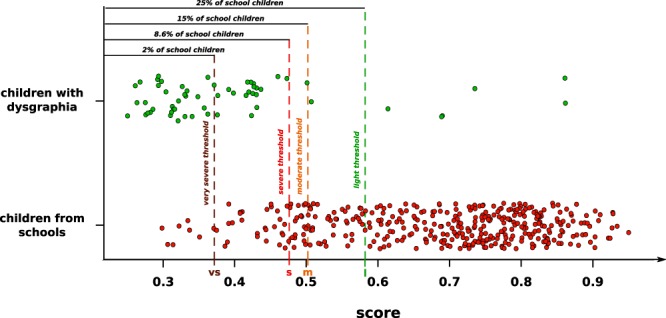
Scores computed for all the children in our database. The red points represent children recruited from schools, while the green points represent children with dysgraphia. Four threshold values (*very severe*, *severe*, *moderate*, *light*) were used to divide handwriting difficulties into five categories with 2% of school children below the *very severe* threshold (this allowed us to compute the *vs* value), 8.6% of school children below the *severe* threshold (this allowed us to compute the *s* value, which is the current dysgraphia threshold), 15% of children below the *moderate* threshold (this allowed us to compute the *m* value) and 25% of children below the *light* threshold (this allowed us to compute the *l* value). It is important to note that these thresholds have been set as examples, meaning that any other values can be used. The score is proportional to the handwriting quality.

One of the way to escape a binary determination (with or without severe handwriting difficulties) with the K-Means clustering algorithm is to exploit the parameters found by the algorithm in order to cluster the data points. The K-means algorithm identifies k cluster centroids (k = 2 in our case) and then allocates each data point to its nearest centroid. In such a manner, the centroid of the cluster grouping typically developing children should represent the handwriting of a *typical* child without handwriting difficulties. The distance between one individual and this centroid location would then be a good indicator of their handwriting quality. In Fig. 4, we can see the scores for both school children and children with dysgraphia. We can see that the scores of school children (0.69 ± 0.13) are statistically higher than the scores of children with dysgraphia (0.41 ± 0.13). A Wilcoxon-Mann-Whitney statistical test (*U* = 1917.0, *p* = 9.67*e* − 25) was used to test statistical significance since the data were not found to be normally distributed. On the basis of the distribution of scores for the school children, we were then able to create threshold values dividing handwriting difficulties into five categories in such a way that: 2% of school children were below the very severe threshold (to find the *vs* threshold), 8.6% of school children were below the severe threshold (to find the *s* threshold) (which is the current dysgraphia threshold), 15% of children were below the moderate threshold (to find the *m* threshold) and 25% of children were below the light threshold (to find the *l* threshold). It should be noted that these thresholds were set as an example, meaning that the number of categories as well as the values of the thresholds defining them can be defined freely.

## Discussion

In this paper, we propose various scales to evaluate handwriting difficulties in a modernized way. These scales exploit the multi-modal data obtained with digital tablets and appear to be disruptive for several reasons.

The current methods used in assessing handwriting difficulties, such as the gold standard test in French- speaking countries (BHK)^19^, are all rule-based systems built on rules designed by a group of experts in handwriting difficulties. Unlike data-driven systems, the consequences of rule-based systems are determined by humans and their subjective knowledge of such complex problems. In addition, a rule-based system will not change or update on its own, which is a limitation, as the educational system and handwriting, in particular, are evolving constantly. In addition, data-driven models are built from the data, meaning that they do not reduce a given problem to a set of “limited” rules extracted from human subjectivity but rather try to model the problem’s complexity with statistical findings coming from factual data.

That being said, it would certainly be a great mistake to neglect years of research and the experience of hundreds of researchers and clinicians interested in this field and build an entirely new handwriting scale. Combining both the advantages of rule-based and data-driven models, our method leans on the crucial expertise of therapists and researchers and exploits machine learning techniques to glean complex and objective knowledge about handwriting difficulties. Indeed, the features used by our model were designed with the help of therapists and therefore aim to capture the current knowledge and rules adept to explain handwriting difficulties. We then combine these rules with a data-driven approach (unsupervised machine learning) to find the handwriting aspects (and their interactions) best explaining the differences in handwriting between children. In such a manner, our data-driven strategy based on multi-modal features provides an objective way to evaluate handwriting difficulties but still captures the current knowledge of experts in this field.

The results provided by our method were then compared with the results of the BHK test conducted on paper. A clear correlation was found since more than 91% of children with dysgraphia (according to the BHK test^19^) were identified with our method, while 87% of the children recruited from schools were assessed as having no handwriting problems. It is interesting to note that 13% of the children recruited in schools were assessed as presenting handwriting difficulties according to our method, corresponding to the 10% of the population in French-speaking countries considered with dysgraphia^19^.

Secondly, our method allows handwriting quality to be measured on a numeric scale, allowing a new categorization of handwriting difficulties. Thanks to our numerical scale, we were able to define new categories according to the level of handwriting difficulties, namely *very severe*, *severe* (corresponding to the current dysgraphia level in French-speaking countries), *moderate* and *light*.

Thirdly, by repeating the process achieved to obtain global handwriting scores but with just the features from a single category (static, pressure, kinematic and tilt), we were able to design new scales evaluating handwriting for these categories. As can be seen in Table 1, if the kinematic (78% of sensitivity) and, to some extent, the pressure (58% of sensitivity) scales appears to be correlated with the overall handwriting difficulties, then neither the static or tilt scale will provide a clear distinction between children with and without handwriting difficulties. This finding shows that the current handwriting tests, which only take the static aspect of handwriting in consideration, are clearly limited since the other aspects of handwriting (kinematic and pressure), which appear to be more important (as can be seen in Fig. 2 and Table 1), are not used. This result also shows that when the handwriting skills (e.g., the pressure or kinematic skills) are considered separately, the results are not as good as when they are considered altogether. For this reason, we believe that a scale assessing handwriting as a combination of skills, such as the one presented in this study, presents added value to therapists and school teachers.

**Figure 5:**
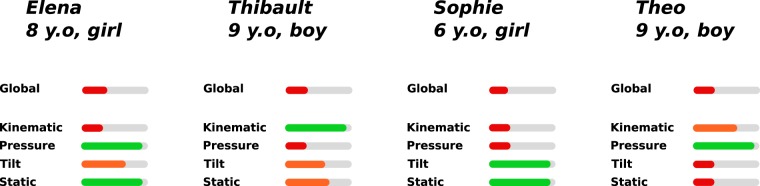
Different handwriting profiles of children with severe handwriting difficulties. We can see that the same condition (dysgraphia) can be expressed in very different ways, with children presenting difficulties in different and independent skills (Kinematic, Pressure, Tilt and Static).

Furthermore, the combination of feature, category and global scores that can be extracted thanks to our method could also be of great use for therapists since handwriting can be assessed according to different aspects and at different granularities (as can be seen in Fig. 5). This flexibility is particularly interesting since the handwriting skills measured by our categories (e.g., kinematic skills, pressure skills, and so on) appear to be quite independent. Hence, measuring any of these skills separately fails to provide a solid measure of a child’s handwriting proficiency (as shown in Table 1). In other words, a child with severe handwriting difficulties does not necessarily have difficulties with all the skills assessed by our categories (as illustrated in Fig. 5). Likewise, a child without handwriting difficulties is not necessarily proficient in all the skills assessed by our categories. Thus, our method allows, thanks to the extraction of a child’s handwriting profile, a deeper analysis of handwriting, making it useful for therapists and school teachers in terms of, for example, making informed decisions as to the type of remediation that should be applied in the case of their patient/student with regards to their specific handwriting problems.

Another great advantage of the scales described in this paper is that they are based on features describing handwriting at a very low level, measuring close to the physiological aspect of handwriting. These features are thus independent of the writing content^21^, meaning that, contrary to the majority of existing tests, the text (and, to some extent, the alphabet ?) used does not matter. In that sense, we can avoid the situation in which over-training biases the test results (children learning the text and not handwriting). As good measures of our features can be obtained in only 30 seconds of data acquisition^21^ (compared to 15 minutes for the BHK test, for example^19^), it is now possible to assess handwriting online and at a very high frequency (several times a week, for instance), making it possible to monitor the progress of a child in a totally new way. It is possible that an application running on a consumer tablet (e.g., an iPad) could conduct cheap, frequent, deep, multidimensional and fast analyses of handwriting as well as suggest remediation activities according to the specific handwriting problems detected and monitor a child’s progress on a weekly basis.

Finally, it is important to understand that the scales presented in this paper assess handwriting based on motor skills only. However, many over factors should be considered when it comes to understanding a human being. In particular, the emotional aspect of a child is not assessed via our method. Our ambition was not to provide a global, comprehensive measure of handwriting but rather to provide a complementary tool for the therapist or school teacher to help them better understand their patient/student according to factors which were previously unexplored.

## Method

### Participants

The present study was conducted in accordance with the Helsinki Declaration. It was approved by the EPFL Ethics Committee (Agreement 043-2019) and conducted with the understanding and written consent of each child’s parents and the oral consent of each child. A ’consent to publish’ was obtained from each child’s parent/guardian (Legally authorised representative of each participant).

A total of 390 typically developing (TD) children were recruited from five primary schools in Haute-Savoie, France. Children ranging in age from five to twelve years of age were recruited from 30 classes. None of these children presented known learning disabilities or neuro-motor disorders.

A total of 58 children presenting severe handwriting difficulties (dysgraphia) were also recruited from therapy centers. Patients of seven grapho-therapists (from the Graphydis Association) located in France were also involved in this study. All of these children were diagnosed dysgraphic (severe handwriting difficulties) according to their BHK scores, previously conducted on paper by therapists.

It is important to note that, unlike to the children recruited from the therapy centers, none of the children recruited in the schools were assessed for dysgraphia with the BHK test, which means that some of these children might present severe handwriting difficulties, as well.

### Data collection

The 448 children (school children and children with dysgraphia) involved in this study were asked to write the first five sentences used in the BHK test on an iPad-based application specifically designed for this purpose (Dynamico application v1.0). This application used the Apple pencil.

The data were collected using Dynamico software (CHILI laboratory EPFL), which recorded the x and y coordinates as well as the pressure, azimuth and altitude angle of the pen at a frequency of 60 Hz. In addition, the gender, age and laterality of the writer were saved.

Based on previous work^21^, we extracted, for each child involved in this study, a set of features describing handwriting based on different aspects and sorted into four main categories (static, kinematic, pressure and tilt). These features were found to successfully predict severe handwriting difficulties in the study published by Asselborn *et al*.^21^. All these features are described in the next section.

### Features extraction

Every feature used in this study is described below:

#### Static features

**Figure 6:**
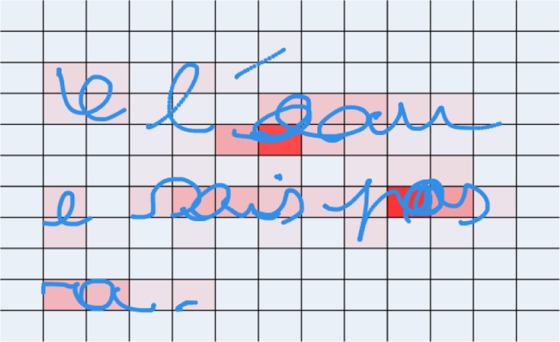
Illustration of the Handwriting density. The space is split into 20-pixel-wide square cells. Using the number of points per cell, we can then compute a density.

**Figure 7:**
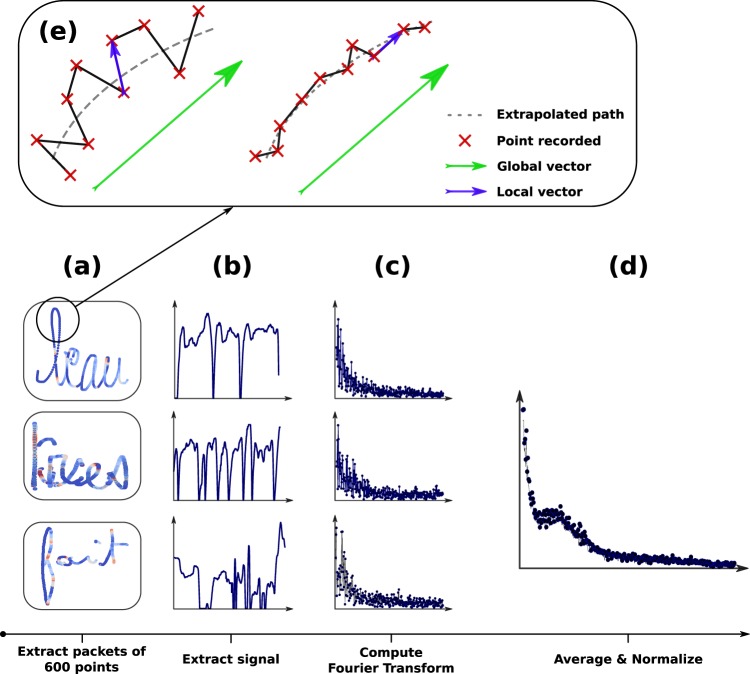
The whole process used to extract the frequency spectrum of our signal. (**a**) We first divided the BHK text into bins of 600 points. (**b**) For each packet, the signal was extracted. (**c**) We computed the Fourier transform of the signal. (**d**) We took the average of all signals and performed a normalization. (**e**) In these sample signals extracted from the data, the red dots are the point coordinates recorded by the device during handwriting, the vectors in blue are “local” vectors linking two consecutive points and the vector in green is the "global” vector (average of the nine blue vectors) representing the global direction of the handwriting. The cross product of these two vectors gives us an indication of the smoothness/shakiness of the handwriting. The right side of the figure comes from a writer with smoother/less shaky handwriting than the writer producing the one on the left, and the cross product operation will detect this difference. This image has been adapted from^21^.

(1) The *Handwriting Moment*. To compute this feature, we extracted bins of 300 points (from the same line of text) and computed their barycenters. The distance in the y direction between consecutive barycenters was computed and averaged for all of the points, reflecting the degree of straightness of the line of text.

(2) The *Handwriting Size*. To compute this feature, we extracted bins of 300 points (from the same line of text) and computed the total surface occupied by the box bounding the trace.

(3) The *Space Between Strokes*. This feature refers to the distance between strokes.

(4) The *Handwriting Density*. A grid with 20-pixel cells covering the entire range of the handwriting trace is created, as can be seen in Fig. 6. The number of points recorded by the iPad in each cell, if present, is then stored in an array. The mean of this array is represented by this feature. There is a positive correlation between handwriting density and handwriting quality, as handwriting becomes denser with age.

(5) The *Average Stoke Direction*. For two consecutive points within a stroke, the direction of movement is calculated as the arctan of the ratio dy/dx. We can obtain the average stroke direction by finding the average of these two directions.

(6) The *Angle Smoothness*. To compute this feature, we first need to calculate the absolute angle between each set of three consecutive points in an handwriting sample. This feature is then obtain by averaging all the angles calculated and measures the smoothness of the handwriting (a high value for this feature relates to abrupt angles).

(7) The *Area of the Text’s Convex Hull*. To compute this feature, we first need to compute the convex hull of the text, which is computed using Graham’s algorithm^31^. We can then compute the area of the convex hull surrounding the text.

(8), (9), (10), (11) (12) The *Bandwidth of the Power Spectral of Tremor Frequencies*, the *Median of the Power Spectral of Tremor Frequencies*, the *Entropy of Mean of Tremor Frequencies*, the *Correlation of Mean of Tremor Frequencies* and the *Distance of Mean of Tremor Frequencies*. These features describe shaky handwriting. For every child, the following process was followed: the signal was first divided into bins of 600 points (as highlighted in Fig. 7). For each bin, we extracted the deviance from the handwriting path using two types of vectors: the first one was a vector representing the global handwriting direction (computed with 10 points, as can be seen in green in Fig. 7), while the second was a local vector computed with two consecutive points (in blue in Fig. 7). We then computed the cross product of these two vectors to calculate how orthogonal the local vectors were with the global vector. The greater this cross product is, the higher the deviance from the handwriting path. We hypothesized that shaky handwriting would result in local vectors that were poorly aligned with the global vectors. Making it possible to detect shaky handwriting with this method.

For each of these 600 points, we saved the norm of the cross product and computed the Fourier transform on this vector. Then we averaged all the Fourier transforms extracted from all the different 600-point bins (see Fig. 7). A high frequency or a wide bandwidth in the spectral distribution would be an indication of shaky handwriting.

For instance, we computed the range of frequencies covering 90% of the spectral density. The smaller this value is (corresponding to a more clustered distribution), the more proficient the writer is. This feature is called the (8) *Bandwidth of the Power Spectral of Tremor Frequencies*. We also extracted the median of the power spectral density. A large value for this feature indicates the presence of high frequencies. This feature is called the (9) *Median of the Power Spectral of Tremor Frequencies*.

The last feature defined in this context is the entropy between the spectral distribution of the writer and the averaged spectral distribution of all the writers in our database. The higher this entropy is, the more different the handwriting of this particular writer is. This feature is called (10) *Entropy of Mean of Tremor Frequencies*. We also computed the correlation between the two signals, called the (11) *Correlation of Mean of Tremor Frequencies*, as well as the distance between them, called the (12) *Distance of Mean of Tremor Frequencies*.

#### Kinematic features

(13), (14) & (15) The *Mean Velocity*, *Maximum Velocity* and *Standard Deviation of Velocity*. These features quantify handwriting speed, where the speed is the distance traveled by the pen divided by the time taken to write out the passage. Research shows that children presenting handwriting difficulties have lower mean velocities as well as higher maximum velocities. Furthermore, the mean velocity increases with age.

(16) The *Increase in Velocity*. Using the handwriting speed over time, we performed a linear regression to compute the evolution of this handwriting speed.

(17) The *Nb of Peaks of Velocity Change Per Second*. Motivated by insights from clinicians, we applied a Gaussian filter to the velocity signal over time (using the scipy library, method *general gaussian* with *M* = 3, *p* = 0.5, *sigma* = 2) and computed the number of local maximum and minimum that were extracted. We expected that the number of changes would grow with the total duration of the test and, therefore, we normalize this number with time.

(18), (19), (20), (21) & (22) The *Bandwidth of the Power Spectral of Speed Frequencies*, the *Median of the Power Spectral of Speed Frequencies*, the *Entropy of Mean of Speed Frequencies*, the *Correlation of Mean of Speed Frequencies* and the *Distance of Mean of Speed Frequencies*. Handwriting can be interpreted as a two-dimensional time series. As in the (19) *Median of the Power Spectral of Tremor Frequencies*, a Fourier transform can be calculated with the handwriting velocity, median and resulting bandwidth of the spectral distribution. We can observe very rapid changes in speed in the handwriting of children with dysgraphia (some jerks resulting from a low level of handwriting automation). These abnormal changes in speed are translated into high frequencies in the Fourier transform, resulting in a shift in the median towards higher frequencies. Children with a low level of automation change also use variable speeds in their writing. Hence, a writer presenting a high bandwidth will not be fluent, as they are less consistent in their movements. The (20) *Entropy of Mean of Speed Frequencies* is the entropy between the spectral distribution of the writer and the average spectral distribution of all the writers in our database. The higher this entropy is, the more eclectic the handwriting of this particular writer is. We also computed the (21) *Correlation of Mean of Speed Frequencies*, which is the correlation between the spectral distribution of the writer and the average spectral distribution of all the writers in our database, as well as the (22) *Distance of Mean of Speed Frequencies*.

(23) The *In-Air-Time Ratio* represents the proportion of time the writer spends without touching the surface of the tablet. This feature has been shown to be positively correlated with handwriting problems^21,23,29^.

#### Pressure features

(24), (25) & (26) The *Mean Pressure*, *Maximum Pressure* and *Standard Deviation of Pressure*. These first features concerning pressure are simply the mean, maximum, and standard deviation of the pressure.

(27), (28) & (29) The *Mean Speed of Pressure Change*, *Max Speed of Pressure Change* and *Standard Deviation of Speed of Pressure Change* are extracted by working with averaged bins of 10 recorded points of pressure and dividing the time spent by the difference between two averaged bins of points. These features are then computed by finding the mean, maximum and standard deviation of all measurements.

(30) The *Increase of Speed of Pressure Change*. Using the changes in the Speed of Pressure change over time, we performed a linear regression to track its evolution.

(31) The *Nb of Peaks of Pressure Change Per Second* computes the number of pressure inversions per second.

(32), (33), (34), (35) & (36) The *Bandwidth of the Power Spectral of Speed of Pressure Change Frequencies*, the *Median of the Power Spectral of Speed of Pressure Change Frequencies*, the *Entropy of Mean of Speed of Pressure Change Frequencies*, the *Correlation of Mean of Speed of Pressure Change Frequencies* and the *Distance of Mean of Speed of Pressure Change Frequencies*. The speed of pressure change can be seen as a time series, and frequencies can be extracted using a Fourier transform. The same process as that described in Fig. 7 is followed to extract these five features.

#### Tilt features

**Figure 8:**
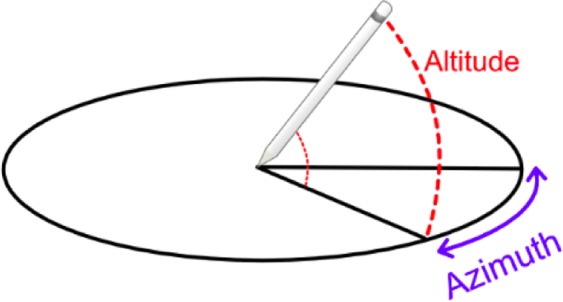
The two angles (Tilt-Azimuth and Tilt-Altitude) recorded for the pen.

The iPad system measures the pen tilt with two different angles referred to as the Tilt-Azimuth angle and the Tilt-Altitude angle (see Fig. 8 for additional information). Both angles are between 0 to 180 degrees.

(38), (39) & (40) The *Mean Tilt-Azimuth*, *Maximum Tilt-Azimuth* and *Standard Deviation of Tilt-Azimuth*. These first features concerning the Tilt-Azimuth are simply its mean, maximum and standard deviation.

(41), (42) & (43) The *Mean Tilt-Altitude*, *Maximum Tilt-Altitude* and *Standard Deviation of Tilt-Altitude*. These first features concerning the Tilt-Altitude are simply its mean, maximum and standard deviation.

(44), (45) & (46) The *Mean Speed of Tilt-Azimuth Change*, *Max Speed of Tilt-Azimuth Change* and *Standard Deviation of Speed of Tilt-Azimuth Change* are extracted by working with averaged bins of 10 recorded Tilt-Azimuth points and dividing the time spent by the difference between two averaged bins of points. These features are then computed by finding the mean, maximum and standard deviation of all measurements.

(47), (48) & (49) The *Mean Speed of Tilt-Altitude Change*, *Max Speed of Tilt-Altitude Change* and *Standard deviation of Speed of Tilt-Altitude Change* are extracted by working with averaged bins of 10 recorded points of Tilt-Altitude and dividing the time spent by the difference between two averaged bins of points. These features are then computed by finding the mean, maximum and standard deviation of all measurements.

(50), (51) The *Increase of Speed of Tilt-Azimuth Change*, *Increase of Speed of Tilt-Altitude Change*. Using the changes in the Speed of Tilt-Azimuth (Tilt-Altitude) over time, we performed a linear regression to track its evolution.

(52), (53) The *Nb of Peaks of Tilt-Azimuth Change Per Second* and *Nb of Peaks of Tilt-Altitude Change Per Second*. This feature computes the number of Tilt-Azimuth (Tilt-Altitude) inversions per second.

(54), (55), (56), (57) & (58) The *Bandwidth of the Power Spectral of Speed of Tilt-Azimuth Change Frequencies*, the *Median of the Power Spectral of Speed of Tilt-Azimuth Change Frequencies*, the *Entropy of Mean of Speed of Tilt-Azimuth Change Frequencies*, the *Correlation of Mean of Speed of Tilt-Azimuth Change Frequencies* and the *Distance of Mean of Speed of Tilt-Azimuth Change Frequencies*. The speed of Tilt-Azimuth change can be seen as a time series, and frequencies can be extracted using a Fourier transform. The same process as that described in Fig. 7 is followed to extract these five features.

(59), (60), (61), (62) & (63) The *Bandwidth of the Power Spectral of Speed of Tilt-Altitude Change Frequencies*, the *Median of the Power Spectral of Speed of Tilt-Altitude Change Frequencies*, the *Entropy of Mean of Speed of Tilt-Altitude Change Frequencies*, the *Correlation of Mean of Speed of Tilt-Altitude Change Frequencies* and the *Distance of Mean of Speed of Tilt-Altitude Change Frequencies*. The speed of Tilt-Altitude change can be seen as a time series, and frequencies can be extracted using a Fourier transform. The same process as that described in Fig. 7 is followed to extract these five features.

## Data Availability

The data that support the findings in this study are available from the corresponding author upon reasonable request.

## Code availability

The code is available from the corresponding author upon reasonable request.
